# Terahertz Time-Domain Spectroscopic Study of Tricalcium Silicate

**DOI:** 10.3390/s22239354

**Published:** 2022-12-01

**Authors:** Wentao Zhang, Ya Chen, Lidan Tian, Linhao Wang, Xiangyu Li

**Affiliations:** College of Civil Engineering, Taiyuan University of Technology, Taiyuan 030024, China

**Keywords:** terahertz time-domain spectroscopy, C_3_S, Beer–Lambert law, effective medium model

## Abstract

In this study, the terahertz (THz) spectra of C_3_S were obtained in the 0.4–2.3 THz frequency range using different sample preparation methods. In the spectra, a sharp absorption peak of C_3_S was found at 2.03 THz. Under controlled conditions, the mass ratio of C_3_S was the most critical factor affecting the strength of the absorption peak, and the absorption coefficient followed the Beer–Lambert law, exhibiting a linear relationship with the mass ratio of C_3_S. The intrinsic dielectric constants of C_3_S and polyethylene (PE) were calculated in accordance with the Maxwell–Garnett (MG), Bruggeman (BM), and Landau–Lifshitz–Loovenga (LLL) models, using two-phase composite samples. The results show that the LLL model had the highest accuracy.

## 1. Introduction

Terahertz (THz) waves are electromagnetic waves in the 0.1–10 THz and 0.03–3 mm frequency and wavelength ranges, respectively [1]. By using THz waves, THz time-domain spectroscopy (THz-TDS) has been developed as a common method for collecting THz spectra with high signal-to-noise ratios. The amplitude and phase information of THz waves transmitted through the sample and, therefore, its optical properties can be obtained using THz-TDS [2]. Studies [3] have shown that protein molecules can be identified through their optical properties, which are obtained from terahertz spectroscopy. The THz technique has also been used to study inorganic minerals. Sakai et al. [4] reported that calcite and aragonite exhibit different absorption coefficients and refractive indices at 3.32 THz in the terahertz spectrum. Hao et al. [5] found that the optical properties (refractive index and absorption coefficient) of natural dolomite change after calcination owing to the phase transformation. THz spectroscopy is a new nondestructive testing technique for understanding the structure of inorganic minerals. In addition, THz spectroscopy can provide information that other spectroscopic methods cannot.

Tricalcium silicate (C_3_S) is the most critical mineral phase of Portland cement clinker, accounting for approximately 60% of the cement mass. The structure of C_3_S significantly impacts cement hydration and, therefore, the properties of cement-based materials [6]. Dolado et al. [7] studied ordinary Portland cement (OPC) and geopolymer (GEO) pastes using THz spectroscopy and conducted molecular simulations to calculate the dielectric response of C–S–H and N–A–S–H gels, which are the most crucial hydration products of OPC and GEO cement pastes. Dash et al. [8] reported an absorption peak of CaCO_3_ at 3.24 THz, and Ray et al. [9] found that Ca(OH)_2_ shows an absorption peak at 9.4 THz. To better understand cement hydration and the properties of cement-based materials, it is important to understand the properties of C_3_S. THz spectroscopy is a novel and alternative noncontact and nondestructive approach for studying the properties of inorganic minerals. However, to the best of our knowledge, the optical properties of C_3_S, such as the absorption coefficient and dielectric constant, have not been studied in the THz frequency range using terahertz spectroscopy.

In this study, the terahertz spectra of C_3_S in the 0.4–2.3 THz range were obtained using a THz-TDS system. Different sample preparation methods were compared for obtaining better spectra. The absorption coefficients of the samples were obtained using THz spectra. The dielectric constants of C_3_S were then extracted and verified using effective medium models.

## 2. Materials and Sample Preparation

### 2.1. C_3_S and PE

**Figure 1:**
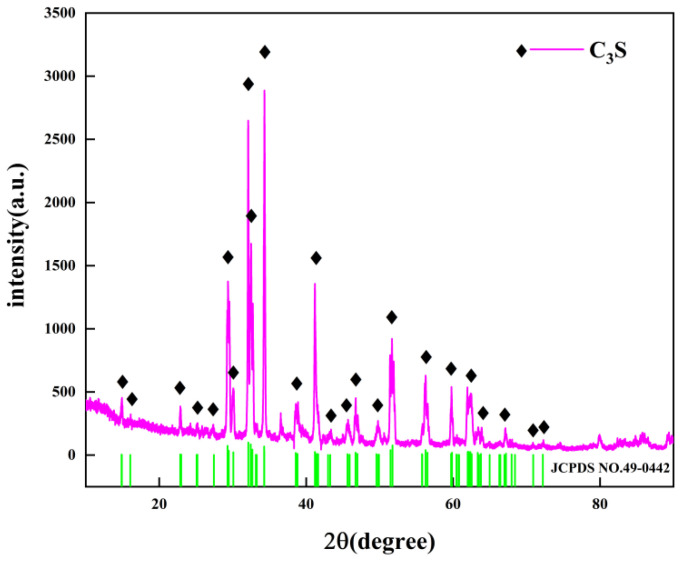
XRD pattern of C_3_S.

**Figure 2:**
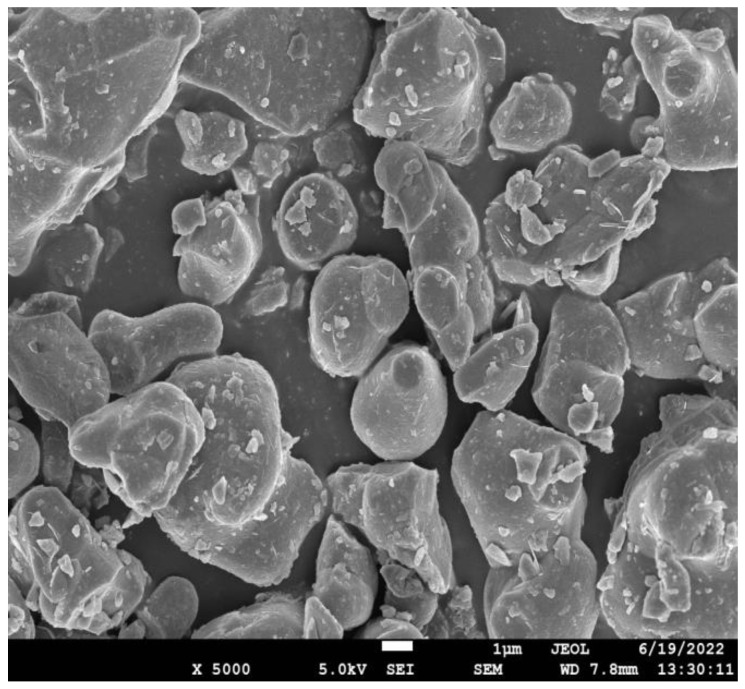
SEM image of C_3_S.

**Figure 3:**
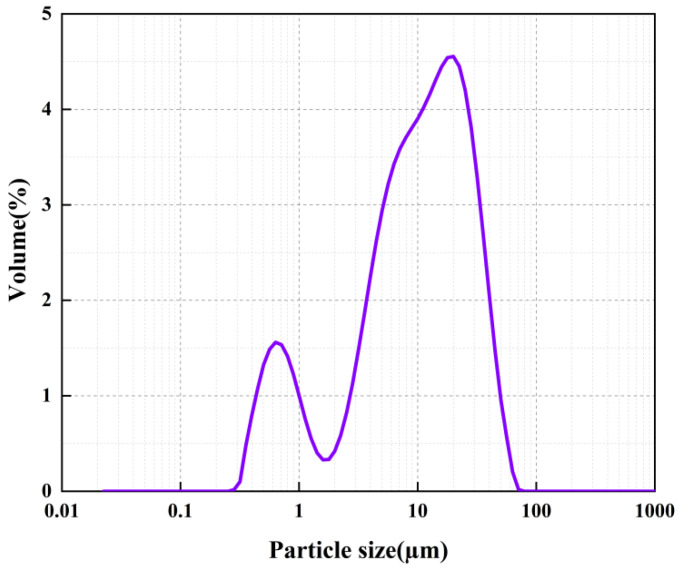
Particle size distribution of tricalcium silicate powder.

The C_3_S used in this study was purchased from Mineral Research Processing (France). Figure 1 shows the X-ray diffraction (XRD) analysis of C_3_S conducted using an X-ray diffractometer (Smartlab, Japan). The data collection range was 5–90° 2θ for CuKα radiation with a step size of 0.02° 2θ. By comparing with standard card No. 49-0442 of C_3_S (the green line in Figure 1), it could be confirmed that the sample represented an almost-pure mineralogical species of the C_3_S mineral. Figure 2 shows the morphology of the C_3_S particles obtained using a scanning electron microscope (ULTIMMAX40, Oxford, UK). The particles had irregular shapes and smooth surfaces. Figure 3 shows the particle size distribution of C_3_S powder measured using a laser particle size analyzer (Mastersizer 2000, Malvern, UK). In THz measurements, severe terahertz scattering can occur within the sample because of large particle size. The average particle size of C_3_S was 9.97 μm and 90% of the particles were smaller than 29.53 μm, which is considerably smaller than the THz wavelengths (130–750 μm) used in the current study. Table 1 shows the specific particle size of C_3_S. Therefore, the scattering effect of particles can be ignored in this experiment [10]. 

In this study, high-density polyethylene (PE) was the shaping material mixed with C_3_S particles to form a disc sample. PE can be considered a THz-transparent material owing to the low absorption of THz waves [11]. It has been widely used for dilution and shaping in THz spectroscopy [12]. 

### 2.2. Sample Preparation

In this study, samples for THz-TDS measurements were prepared by mixing C_3_S particles with PE. After mixing, the mixture was pressed to form a small disc. The compression force and mass ratio between C_3_S and PE can affect THz measurements. Therefore, to obtain reliable and accurate results, we investigated the influences of different sample preparation variables on the THz spectrum and, hence, the optical properties. Four sample groups were prepared.

Group_1 had four different samples prepared by disc-pressing different amounts of C_3_S particles separately. The masses of the four samples were 150, 200, 250, and 300 mg. The C_3_S particles were pressed for 3 min at a pressure of 10 MPa within a die with an internal diameter of 13 mm. The surfaces of the samples were even and smooth, and no cracks were observed. 

Group_2 had three samples prepared by disc-pressing the same amount (300 mg) of C_3_S particles at different pressures. The pressures of the three samples were 6, 10, and 15 MPa. The pressure was maintained for 3 min for all three samples. 

Group_3 had three samples prepared by disc-pressing the same amount (300 mg) of C_3_S particles with the same pressure (10 MPa), but for different holding times. The pressure holding times for the three samples were 3, 4, and 5 min. It should be noted that Group_1, 2, and 3 samples were two-phase composites since they contained C_3_S and pores.

Group_4 had 11 samples prepared by disc-pressing PE and C_3_S particles in different mass ratios. The pressure was 10 MPa, and the holding time was 3 min for all samples. The total mass of each sample was 150 mg, and the mass ratio of C_3_S was varied from 0–100% in steps of 10%. Group_4 samples were three-phase composites because they contained C_3_S, PE, and pores.

## 3. THz-TDS System and Data Analysis

**Figure 4:**
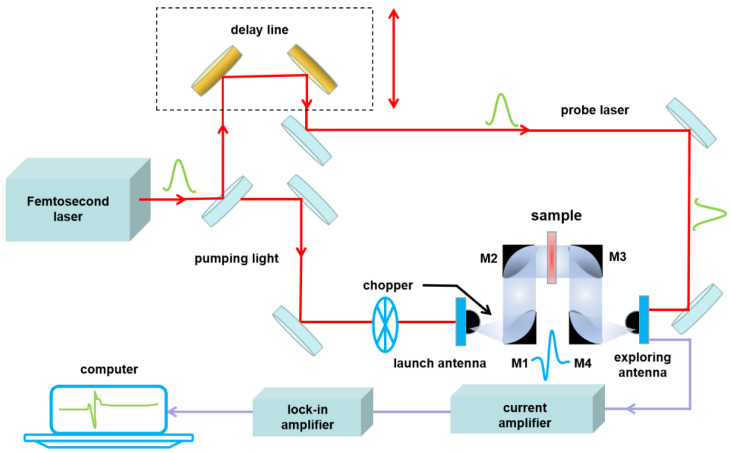
Schematic drawing of THz-TDS system.

The THz experiment used a THz-TDS system (TAS 7500SU, Advantest, Japan) from LET Terahertz Technology Co., Ltd. (Tianjin, China) with a 0.2–7 THz frequency range, a 7.6 GHz frequency resolution, and a 57 dB dynamic range. Figure 4 shows the THz-TDS system, including a femtosecond laser, terahertz transmitting antenna, and receiving antenna. It should be noted that, in the current study, we analyzed a 0.4–2.3 THz signal since this frequency range had the highest signal-to-noise ratio.

The data acquisition process of the THz time-domain signal of the sample was as follows: first, the reference signal was collected when there was no sample in the optical path, and then the disc sample was placed to collect the sample signal for three readings. The experimental results of the three measurements were averaged to exclude sampling and systematic error. During the experiment, to avoid water vapor, which has a strong influence due to its strong absorption of THz waves in air, nitrogen gas was continuously filled in the interior of the device to expel water vapor [13]. During the experiment, the ambient temperature was maintained at 22 °C.

The frequency-domain signal of the samples can be obtained by the fast Fourier transform of the time-domain reference signal Er(t) and sample signal Es(t). The optical properties, including the refractive index $n\left(\omega \right)$, absorption coefficient $\alpha \left(\omega \right),$ and extinction coefficient $k\left(\omega \right)$ can be derived using the method proposed by Duvillaret et al. [14] as follows:

(1) $$n\left(\omega \right)=\frac{c\Delta \varphi }{\omega d}+1,$$

(2) $$\alpha \left(\omega \right)=\frac{2}{d}{\left(\ln\frac{4n\left(\omega \right)}{{\left[\rho \left(\omega \right)\left[n\left(\omega \right)+1\right)\right]}^{2}]}\right)}^{2},$$

(3) $$k\left(\omega \right)=\frac{c\alpha \left(\omega \right)}{2\omega }=\frac{c}{d\omega }\ln\left[\frac{4n\left(\omega \right)}{{\left(n\left(\omega \right)+1\right)}^{2}\rho \left(\omega \right)}\right],$$

(4) $$\frac{E_{r}\left(\omega \right)}{E_{s}\left(\omega \right)}=\rho \left(\omega \right)\cdot e^{-\varphi \left(\omega \right)},$$

(5) $$\varepsilon \left(\omega \right)=n{\left(\omega \right)}^{2}-k{\left(\omega \right)}^{2},$$

where ω denotes the angular frequency, Δφ denotes the difference between the phase of the sample signal and reference signal after the fast Fourier transform, d denotes the thickness of the sample, $k\left(\omega \right)$ denotes the extinction coefficient of the piece, $\rho \left(\omega \right)$ denotes the transmission coefficient of the piece, and $\varepsilon \left(\omega \right)$ denotes the dielectric constant of the piece.

## 4. Effective Medium Theory

Effective medium theory (EMT) has been widely used to study the effective macroscopic properties of multiphase materials. A range of effective medium models have been developed on the basis of the effective medium theory. These models can be used to calculate the macroscopic properties of multiphase materials by using the properties of each phase and its volume fraction [15]. The most frequently used effective medium models include the Maxwell–Garnett (MG), Bruggeman (BM), and Landau–Lifshitz–Loovenga (LLL) models [16,17].

### 4.1. Maxwell–Garnett (MG) Model

The MG model was developed by assuming that the multiphase material consists of a host material embedded with small spherical guest materials. Taking the dielectric constant of a multiphase material as an example, the equation for the MG model is as follows:

(6) $$\frac{\varepsilon_{\mathrm{MG}}-\varepsilon_{h}}{\varepsilon_{\mathrm{MG}}+2\varepsilon_{h}}=\overset{N}{\underset{n=1}{\sum }}f_{n}\frac{\varepsilon_{n}-\varepsilon_{h}}{\varepsilon_{n}+2\varepsilon_{h}},$$

where $\varepsilon_{h}$ is the dielectric constant of the host material, $\varepsilon_{n}$ is the dielectric constant of the n-th guest material, $\varepsilon_{\mathrm{MG}}$ is the effective (aggregate) dielectric constant of the multiphase material, and $f_{n}$ is the volume fraction of the n-th guest material. 

It should be noted that the MG model is valid for multiphase materials with a lower volume fraction (usually less than 30%) [18] of the guest material and a particle size significantly smaller than the spacing between them. In addition, the MG model is asymmetric in the exchange of the host and guest phases.

### 4.2. Bruggeman (BM) Model

The BM model is an improved version of the MG model, as shown in Equation (7). The particle shape of the guest material is assumed to be small spheres. Compared to the MG model, the volume fraction of the guest materials is elevated because the BM model is symmetric with regard to switching the host and guest phases. Therefore, the BM model is expected to be more accurate when the volume fraction of the guest phase is larger [19]. The equation for the BM model is as follows:

(7) $$\overset{N}{\underset{n=1}{\sum }}f_{n}\frac{\varepsilon_{n}-\varepsilon_{\mathrm{BM}}}{\varepsilon_{n}+2\varepsilon_{\mathrm{BM}}}=0.$$

### 4.3. Landau–Lifshitz–Loovenga (LLL) Model

For the LLL model, the effective dielectric constant of a multiphase material is modeled as follows:

(8) $$\varepsilon_{\mathrm{LLL}}{}^{1/3}=\overset{N}{\underset{n=1}{\sum }}f_{n}\varepsilon_{n}{}^{1/3}.$$

The LLL model is also symmetric with regard to the exchange of the host and guest phases. Unlike the MG and BM models, the LLL model allows guest material to have any shape. In addition, the LLL model was derived by assuming that the multiphase material is a low-dielectric-contrast composite. Scheller et al. [20] validated the LLL model using a mixture of calcium carbonate and polypropylene with a dielectric contrast of 6.63.

## 5. Results and Discussions

### 5.1. THz Absorption Spectra of Different Samples

**Figure 5:**
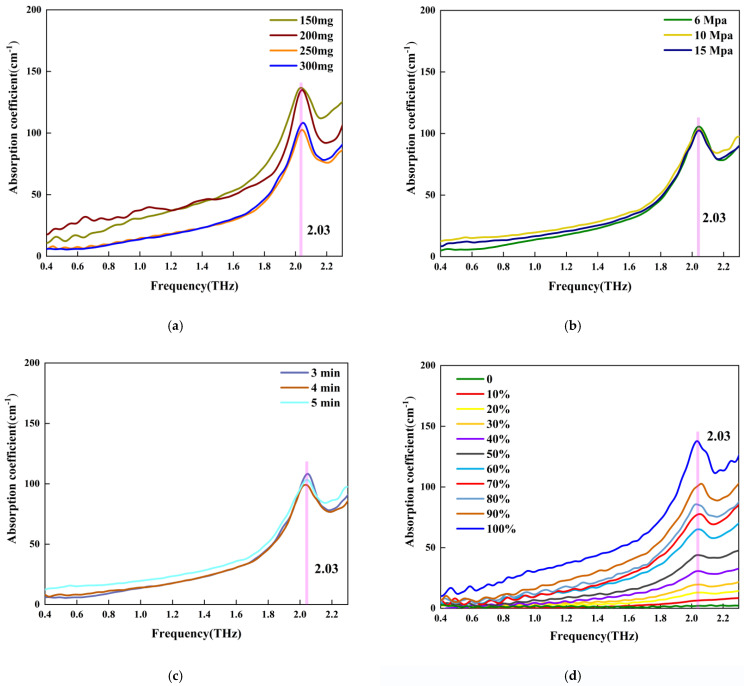
THz absorption spectra of four groups samples. (**a**) Absorption spectra of Group 1 samples. (**b**) Absorption spectra of Group 2 samples. (**c**) Absorption spectra of Group 3 samples. (**d**) Absorption spectra of Group 4 samples.

Figure 5 shows the THz spectra of four groups of samples prepared under different conditions. As shown, there was a sharp absorption peak at 2.03 THz for the sample containing C_3_S. This could be useful for studying the structure of C_3_S and its transformation during cement hydration. However, the assignment of the peak needs to be studied, hopefully in future theoretical research.

Figure 5a shows the THz absorption spectra of Group_1 samples. As can be seen, the absorption coefficients of the C_3_S disc sample increased with an increase in frequency. There was an absorption peak located at 2.03 THz for all the samples. The absorption peaks of the four samples were very sharp, and their peak shape was very similar. This is probably because they had the same mass ratio of C_3_S, i.e., 100%. However, there was a difference in their absorption coefficients, potentially due to their different thickness [12].

Figure 5b shows the THz absorption spectra of Group_2 samples. The pressure had no impact on the absorption spectra. This means that, in the current study, it was sufficient to prepare a THz sample using 6 MPa and 3 min. Figure 5c shows the influence of the pressure holding time on the absorption spectra. As can be seen, the absorption spectra were almost identical for all three samples. Therefore, when the pressure was 10 MPa, 3 min was sufficient to prepare the THz sample. Figure 5d shows the absorption spectra of the samples containing both C_3_S and PE. It can be seen that the absorption peak strength at 2.03 THz increased with an increase in C_3_S content in the sample. However, it would be better to contain at least 30% C_3_S in the mixture sample for detection.

**Figure 6:**
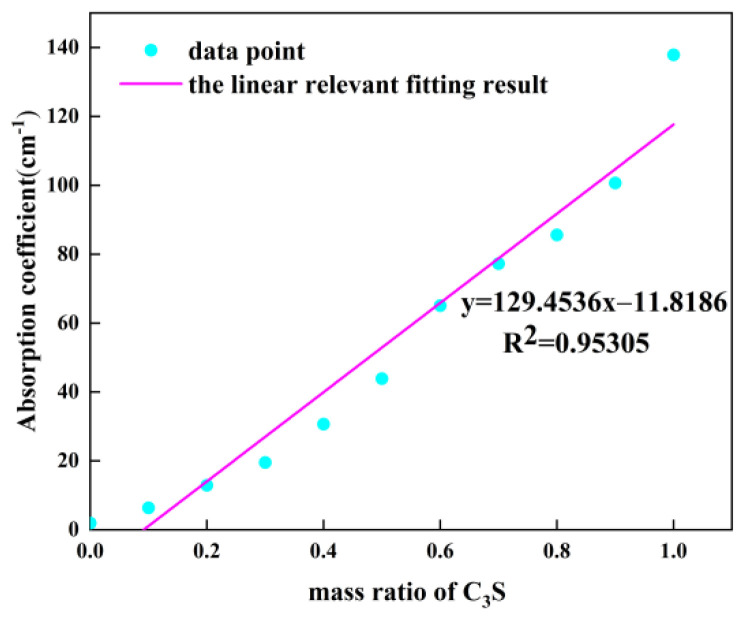
Linear relationship between C_3_S content and absorption coefficient.

Figure 6 plots the relationship between the absorption coefficient at 2.03 THz and C_3_S content in the sample. As shown, the relationship between the absorption coefficient and C_3_S content in the sample can be predicted using the Beer–Lambert law [21].

### 5.2. THz Dielectric Spectra of Two-Phase Composite Samples

Dielectric spectra were obtained for two types of samples, i.e., the C_3_S and PE samples. One type of sample was prepared by disc-pressing only C_3_S particles.

**Figure 7:**
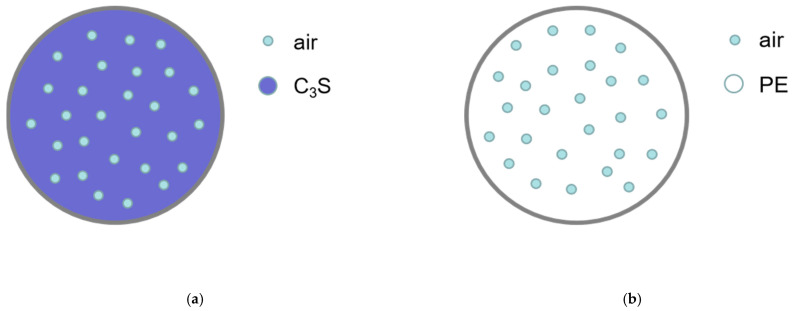
Schematic drawing of C_3_S and PE disc samples: (**a**) C_3_S disc; (**b**) PE disc.

The other type was fabricated by disc-pressing only PE; these samples were two-phase composites. The C_3_S sample contained both air and C_3_S. The PE samples contained PE and air. Figure 7 shows a schematic of the two types of samples. By utilizing the EMT and THz dielectric spectra, we calculated the intrinsic dielectric constants of PE and C_3_S, respectively. Since the densities of PE and C_3_S are known to be 0.94 g/cm^3^ and 2.61 g/cm^3^, respectively, the volume fraction of each phase in the sample could be calculated.

**Figure 8:**
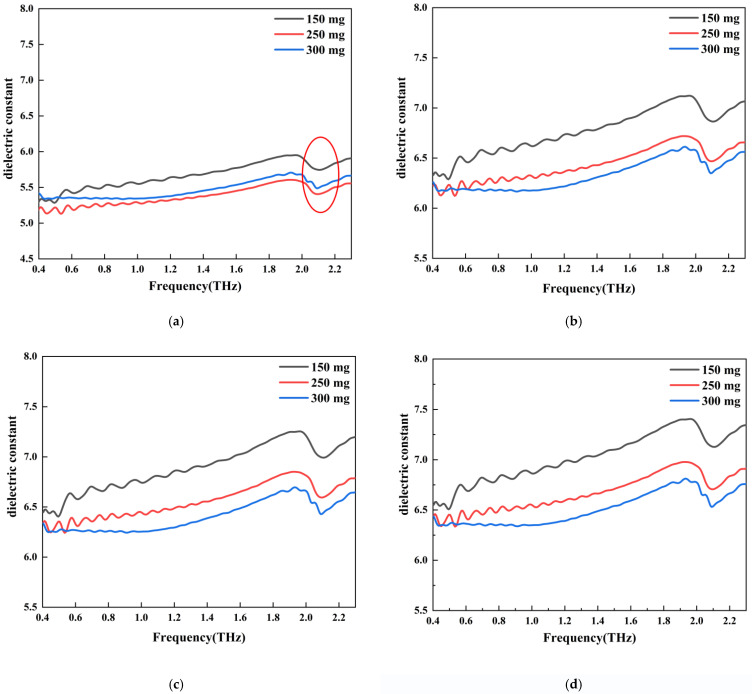
THz dielectric spectra of C_3_S samples with different mass obtained from the experimental test and the intrinsic dielectric spectrum processed by the model: (**a**) C_3_S dielectric constant spectrum (the red circle shows dielectric dispersion in 2.03THz); (**b**) MG model C_3_S intrinsic dielectric; (**c**) BM model C_3_S intrinsic dielectric constant; (**d**) LLL model C_3_S intrinsic dielectric constant.

Figure 8 shows the THz dielectric spectra of the C_3_S samples obtained from THz measurements and EMT calculations. Three samples were tested, with the only difference between them being the mass of C_3_S. As shown in Figure 8a, the dielectric constant of the 150 mg sample was higher than that of the other two samples (250 mg and 300 mg). Dielectric dispersion [22,23,24] was observed near the absorption peak (2.03 THz) for all three samples. As shown in Figure 8a, the dielectric constant increased until 2.03 THz, then started to decrease, and then increased again. For every C_3_S sample, we calculated the intrinsic dielectric constant of the C_3_S mineral using the EMT models. Then, we replotted the THz dielectric spectra of these three samples, as shown in Figure 8b–d, using three models. It can be seen that the calculated THz dielectric spectra followed the same trend as the measured spectra. However, the dielectric constants were all larger than the measured ones. In accordance with the three models, the intrinsic dielectric constants of C_3_S were 6.51, 6.61, and 6.73. It should be noted that the values were averaged in the 0.4–2.3 THz range.

**Figure 9:**
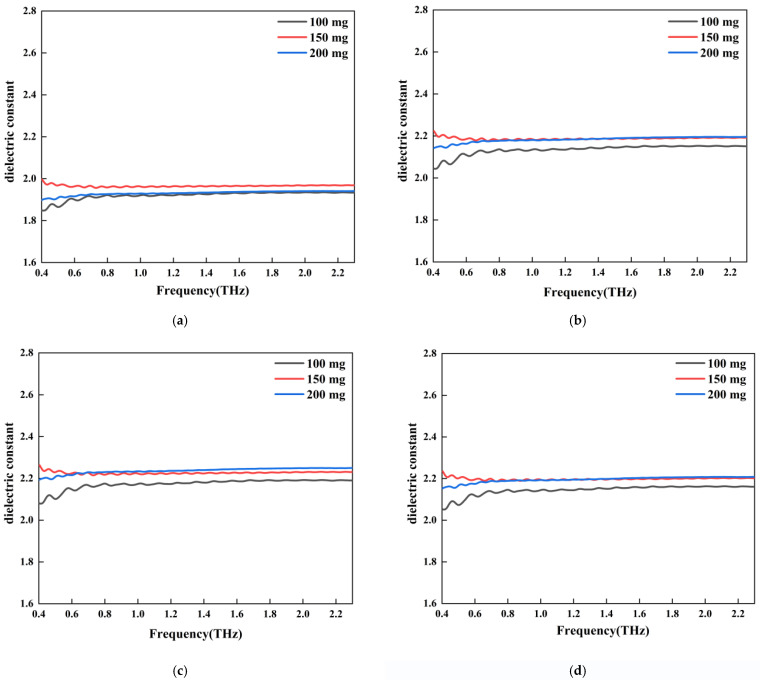
The PE dielectric spectrum obtained from the experimental test and the intrinsic dielectric spectrum processed by the model: (**a**) PE dielectric constant spectrum; (**b**) MG model PE intrinsic dielectric constant; (**c**) BM model PE intrinsic dielectric constant; (**d**) LLL model PE intrinsic dielectric constant.

Figure 9 shows the THz dielectric spectra of PE samples obtained from the THz measurement and EMT calculation. It should be noted that no PE samples exhibited dielectric dispersion since PE absorbs nearly no THz waves. We also replotted the THz dielectric spectra by using measured spectra and EMT models. As shown in Figure 9b–d, the calculated spectra were similar to their measured counterparts except the values were larger. The intrinsic dielectric constants of PE were calculated as 2.17, 2.16, and 2.17, using three EMT models.

### 5.3. THz Dielectric Spectra of Three-Phase Composite Samples

**Figure 10:**
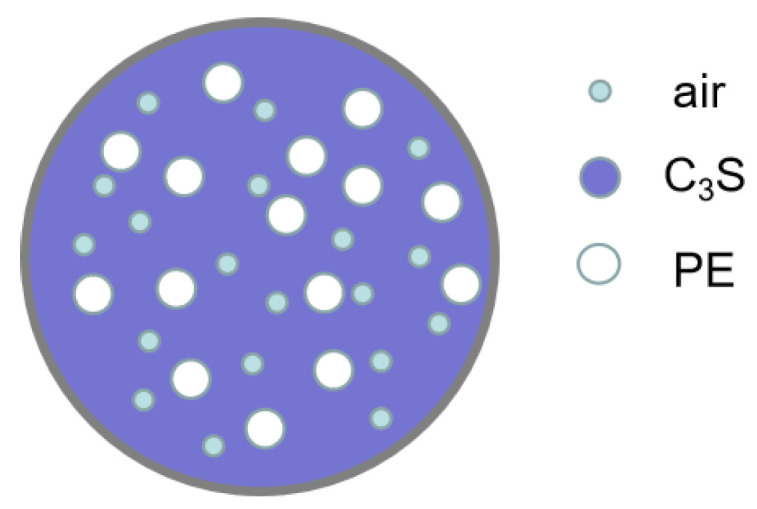
Schematic drawing of three-phase sample.

**Figure 11:**
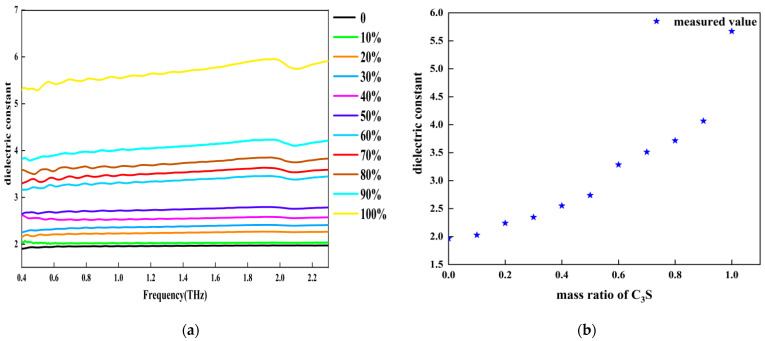
Dielectric constant of a mixture of C_3_S and PE: (**a**) experimental measurement of dielectric spectra of mixtures; (**b**) average dielectric constant in the 0.4–2.3 THz range.

Three-phase composite samples were prepared by disc-pressing C_3_S and PE. The samples were considered three-phase composites due to the pores formed by trapped air. Figure 10 shows a schematic of the three-phase samples. Figure 11 shows the THz dielectric spectra of the samples with various mass ratios of C_3_S ranging from 0% to 100%. The dielectric constants of the samples increased with increasing C_3_S content. This is reasonable because C_3_S has a larger dielectric constant than PE. The THz dielectric constant for every sample can be calculated by taking an average of the dielectric constants from 0.4–2.3 THz. Figure 11 shows the relationship between the mass ratio of C_3_S and the average dielectric constant of each sample. It is apparent that the dielectric constant increased monotonically with increasing C_3_S content in the sample.

**Figure 12:**
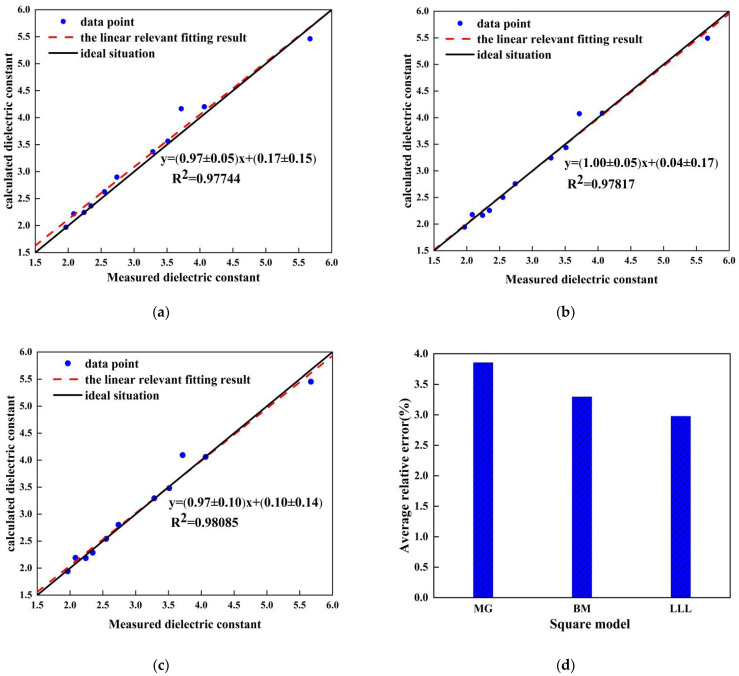
Comparisons of measured and calculated dielectric constants: (**a**) Measured dielectric constant and calculated values of MG model; (**b**) measured dielectric constant and calculated values of BM model; (**c**) measured dielectric constant and calculated values of the LLL model; (**d**) error analysis of each model.

To verify the experimental results, we used three EMT models to calculate the dielectric constant of each sample. The intrinsic dielectric constants of C_3_S and PE were 6.51, 6.61, and 6.73, and 2.17, 2.16, and 2.17, respectively, as determined in Section 5.2. Figure 12 shows comparisons between the measured dielectric constant and the calculated dielectric constant using the three models. As shown in Figure 12a–c, the calculated values agreed with the measured values. The R-squared values for the MG, BM, and LLL models were 0.9774, 0.9782, and 0.9809, respectively. Using Equation (9), we calculated the relative errors, as shown in Figure 12d, to be 3.86%, 3.30%, and 2.98% for the MG, BM, and LLL models, respectively. Therefore, the LLL model was the best for fitting the THz dielectric constants of the samples. Both the MG and the B models were derived by assuming that the guest phase was spherical. However, the LLL model did not have this limitation. In addition, this model is suitable for low-dielectric-contrast composites because it employs Taylor approximation [20]. The dielectric contrast between C_3_S and air is relatively small, which may explain why the LLL model performed better than the other two models.

(9) $$\mathrm{Relative}\;\mathrm{error}=\frac{\sum_{i=1}^{n}│\frac{\varepsilon_{\mathrm{measure},i}-\varepsilon_{\mathrm{calculate},i}}{\varepsilon_{\mathrm{measure}}}│}{N}\times 100\%$$

## 6. Conclusions

In this study, the spectral responses of C_3_S and PE in the 0.4–2.3 THz frequency range were obtained using a THz-TDS system, and a dielectric model was proposed on the basis of the Maxwell–Garnett (MG), Bruggeman (BM), and Landau–Lifshitz–Loovenga (LLL) models to consider the effect of trapped air in the samples. The intrinsic dielectric constants of C_3_S and PE were obtained under different models by considering the influence of air on the dielectric constants in the samples using two-phase effective-medium models. The calculated values were compared with the measured terahertz dielectric constants of the composites. The results showed that the relative errors of MG, BM, and LLL were 3.86%, 3.30%, and 2.98%, respectively, whereby the LLL model had the highest accuracy. The dielectric constant is the physical property of materials, and it is very important to accurately obtain the dielectric constant of cement minerals by terahertz spectroscopy to understand their structure-property relationship. The origin of the absorption peak at THz range can be assigned as the vibration and interactions of molecules. However, the density functional theory calculations need to be conducted for theoretically assigning the vibrational peak.

## Figures and Tables

**Table 1:** Particle size of C_3_S.

Concentration	Span	D [4, 3]	D [3, 2]	d (0.1)	d (0.5)	d (0.9)
0.0074%	2.864	13.156 μm	3.009 μm	0.81 μm	9.972 μm	29.526 μm

## Data Availability

Not applicable.
